# Planning combinatorial disulfide cross-links for protein fold determination

**DOI:** 10.1186/1471-2105-12-S12-S5

**Published:** 2011-11-24

**Authors:** Fei Xiong, Alan M Friedman, Chris Bailey-Kellogg

**Affiliations:** 1 Department of Computer Science, Dartmouth College, Hanover, NH 03755, USA; 2 Department of Biological Sciences, Markey Center for Structural Biology, Purdue Cancer Center, and Bindley Bioscience Center, Purdue University, West Lafayette, IN 47907, USA

## Abstract

**Background:**

Fold recognition techniques take advantage of the limited number of overall structural organizations, and have become increasingly effective at identifying the fold of a given target sequence. However, in the absence of sufficient sequence identity, it remains difficult for fold recognition methods to always select the correct model. While a native-like model is often among a pool of highly ranked models, it is not necessarily the highest-ranked one, and the model rankings depend sensitively on the scoring function used. *Structure elucidation* methods can then be employed to decide among the models based on relatively rapid biochemical/biophysical experiments.

**Results:**

This paper presents an integrated computational-experimental method to determine the fold of a target protein by probing it with a set of planned disulfide cross-links. We start with predicted structural models obtained by standard fold recognition techniques. In a first stage, we characterize the fold-level differences between the models in terms of topological (contact) patterns of secondary structure elements (SSEs), and select a small set of SSE pairs that differentiate the folds. In a second stage, we determine a set of residue-level cross-links to probe the selected SSE pairs. Each stage employs an information-theoretic planning algorithm to maximize information gain while minimizing experimental complexity, along with a Bayes error plan assessment framework to characterize the probability of making a correct decision once data for the plan are collected. By focusing on overall topological differences and planning cross-linking experiments to probe them, our *fold determination* approach is robust to noise and uncertainty in the models (e.g., threading misalignment) and in the actual structure (e.g., flexibility). We demonstrate the effectiveness of our approach in case studies for a number of CASP targets, showing that the optimized plans have low risk of error while testing only a small portion of the quadratic number of possible cross-link candidates. Simulation studies with these plans further show that they do a very good job of selecting the correct model, according to cross-links simulated from the actual crystal structures.

**Conclusions:**

Fold determination can overcome scoring limitations in purely computational fold recognition methods, while requiring less experimental effort than traditional protein structure determination approaches.

## Introduction

**Figure 1:**
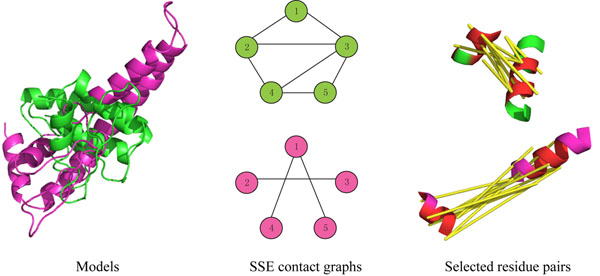
**Protein fold determination by disulfide cross-linking.** The example shows two models, but the method readily handles tens or even hundreds of models. (left) Two models, TS125_3 (green) and TS194_2 (magenta), for CASP target T0351, are of reasonable quality but have rather different topologies. (middle) The three-dimensional structures are compiled into graphs on the secondary structure elements (SSEs), representing the topology in terms of contacting SSE pairs. A topological fingerprint is selected based on differences in SSE contacts (e.g., 1-2, 2-4, 3-5, etc.) that together distinguish the models. (right) For each SSE pair in the topological fingerprint, a set of residue pairs is selected for disulfide cross-linking, in order to robustly determine whether or not the SSE pair is actually in contact. The figure shows the selected cross-links (yellow) to test for SSE pair (1, 2). Residues selected for cross-linking are colored red.

Despite significant efforts in structural genomics, the vast majority (> 90% [1]) of available protein sequences do not have experimentally determined three-dimensional structures, due to experimental expense and limitations (e.g., lack of crystallizability). At the same time, since structure is more conserved than sequence, there may be only a small number (a thousand or two [2,3]) of distinct natural “folds” (overall structural organizations), and many of them can already be found in the protein databank (PDB). Fold recognition techniques [1,4] take advantage of this, and have become increasingly effective at identifying the fold of a given target sequence. However, the series of Critical Assessment of Structure Prediction (CASP) [5] contests demonstrates that, in the absence of sufficient sequence identity, it remains difficult for fold recognition methods to always select the correct model. While a native-like model is often among a pool of highly ranked models, it is not necessarily the highest-ranked one, and the model rankings depend sensitively on the scoring function used [5,6]. Fig. 1(left) illustrates two possible alternative models for one target from a recent CASP competition.

Seeking to close the gap between computational structure prediction and experimental structural determination, we [7,8] and others [9-11] have developed methods (which we call *structure elucidation*) to select structural models based on relatively rapid biochemical/biophysical experiments. One type of experiment particularly suitable for this purpose is *cross-linking*, which essentially provides distance restraints between specific pairs of residues, based on the formation (or not) of chemical cross-links. While residue-specific (e.g., lysine-specific) cross-linking has been effectively used for this task [10,12,13], we previously showed that planned *disulfide* cross-linking has a number of advantages, in terms of the ease and reliability of experiment and the quality of the resulting information content [7]. In disulfide cross-linking (or “trapping”) [14-16], a pair of cysteine substitutions is made and the formation of a disulfide bond after oxidation is evaluated, e.g., by alteration in electrophoretic mobility [7,14,16]. An important point for our purposes here is that disulfide cross-links are *plannable*—we control exactly which pair of residues is probed in a particular experiment.

While earlier methods have focused on probing geometry and selecting a model, we target here a more defined characterization of protein structure, ascertaining the overall protein fold. We call this approach *fold determination*, named in contrast to purely computational *fold recognition* and our less defined structure elucidation approach. We first characterize the topological / fold-level differences in a set of models in terms of contact patterns of secondary structure elements (SSEs); see the middle panel of Fig. 1. The topological representation allows for a robust experimental characterization of the structure, less sensitive to noise and uncertainty in both the models (e.g., threading misalignment) and the actual structure (e.g., flexibility). As a representation with fewer degrees of freedom than the complete threading models, the topological representation also enables us to explicitly consider all possibilities and handle the case when none of the models is correct. Once we have identified a subset of SSE pairs that are most informative for fold determination, we plan disulfide cross-links to evaluate these SSE pairs; see the right panel of Fig. 1. By specifically planning for each such SSE pair, we can account for the dependence among the cross-links and select a set that will be robust to, and even help characterize, model misalignment and protein flexibility.

The method presented here strikes a balance between very limited cross-linking (e.g., six disulfide pairs in our earlier work [7]) and testing all residue pairs. We assume that robotic genetic manipulation methods (e.g., based on SPLISO [17] and RoboMix [18]) can construct a combinatorial set of dicysteine mutants, but that we still should test a much smaller set than all residue pairs. (Our plans require tens to around a hundred cross-links, depending on error requirements.) Thus we must optimize a plan so as to maximize information gain while minimizing experimental complexity. This is analogous to feature subset selection, where the goal is to choose a subset of features from a dataset such that the reduced set still keeps the most “distinguishing” characteristics of the original [19,20]. At the topological level (Fig. 1, middle) the features are SSE pairs, and the objective is to select those that will correctly classify the real structure to a model. At the cross-link level (Fig. 1, right panel) the features are potential disulfide pairs and the objective is to select those that will correctly classify contact/not for the SSE pair. For each level, we optimize a plan by employing an information-theoretic planning algorithm derived from the minimum redundancy maximum relevance approach [21]. We then evaluate a plan with a Bayes error framework that characterizes the probability of making a correct decision from the experimental data.

## Methods

We are given a set *M* of models. They may be redundant (i.e., some may have the same fold), and they may be incomplete (i.e., a representative of the correct fold may not be included). Our goal is to plan a set of disulfide cross-linking experiments (i.e., identify residue pairs to be individually tested) in order to select among them. As discussed in the introduction, we do this in two stages (Fig. 1), first selecting a “topological fingerprint” of SSE pairs to distinguish the folds, and then selecting cross-links to assess these SSE pairs.

### Topological fingerprint selection

In order to compare SSE topologies, we need a common set of SSEs across the models. Since secondary structure prediction techniques are fairly stable [22,23], it is generally the case that models have more-or-less the same set of SSEs, covering more-or-less the same residues (> 50% overlapping as observed in our test data). Our approach starts with a set *S* of SSEs that are common to at least a specified fraction (default 50%) of the given models. For example, both models in Fig. 1 have 5 *α*-helices, as do 63 other models for the same target. The later cross-link planning stage will account for the fact that the common SSEs may in fact extend over slightly different residues in the different models.

Given the SSE identities, we form for each model *m_i_* ∈ *M* an *SSE contact graph G_SSE_*_,_*_i_* = (*S*, *C_i_*) in which the nodes *S* are the SSEs (common to the specified fraction of models, as described in the preceding paragraph) and the edges *C_i_* ⊂ *S* × *S* are between contacting SSEs (specific to each model). We determine SSE contacts from residue contacts, deeming an SSE pair to be in contact if a sufficient set of residues are. Our current implementation requires at least 5 contacts (at < 9 Å C*^β^*-C*^β^* distance), and at least 20% of each SSE’s residues to have a contact partner in the other SSE.

Our goal then is to find a minimum subset *F* ⊂ *S* × *S* of SSE pairs providing the maximum information content to differentiate the models. As discussed in the introduction, this is much like feature subset selection; in particular, the *max-dependency* feature selection problem seeks to find a set of features with the largest dependency (in term of mutual information) on the target class (here, the predicted structural model) [21]. While max-dependency leads to the minimum classification error, there is unfortunately a combinatorial explosion in the number of possible feature subsets that must be considered. To deal with the combinatorial explosion, we develop here an approach based on the minimum Redundancy Maximum Relevance (mRMR) method [21].

#### Probabilistic model

First we develop a probabilistic model in order to evaluate the information content in a possible experiment plan. Let us treat each edge as being a binary random variable c representing whether or not the SSE pair is in contact, with Pr(*c*) the probability of being in contact (*c* = 1) or not (*c* = 0). We estimate Pr(*c*) by counting occurrence frequencies over the contact edge sets *C_i_* for the models:(1)

where the summed variables range over {0, 1} and the indicator function 1 tests for membership of c in set *C_i_*, and thus the set includes those SSE contact graphs for which the contact state of c agrees with *y*. To allow for noise, when evaluating *x* = 1 we include a contribution from *y* = 0 (false negative) along with that for *y* = 1 (true positive), and similarly when evaluating *x* = 0 we consider both *y* = 1 (false positive) and *y* = 0 (true negative). The *q* function weights the contributions for the agreeing and disagreeing case. We currently employ a uniform weighting independent of edge, since we observed in cross-link planning (below) that the expected error rate in evaluating any SSE contact was well below 10% when using a reasonable number of cross-links:(2)

The approach readily extends to be less conservative and to allow different weights for different SSE pairs, e.g., according to cross-link planning (discussed in the next section).

We can likewise compute a joint probability Pr(*c*, *c*′) from co-occurrence frequencies:(3)

where again the sums are over {0, 1} and the indicator function is as described above.

Then we can evaluate the *relevance* of each SSE contact edge c in terms of its entropy *H*(*c*); a high-entropy edge will help differentiate models while a low-entropy one won’t. We can also evaluate the *redundancy* of a pair (*c*, *c*′) of edges in terms of their mutual information *I*(*c*, *c*′); a high mutual-information pair contains redundant information:(4)(5)

#### Experiment planning

The mRMR approach seeks to minimize the total mutual information (redundancy) and maximize the total entropy (relevance). In this paper, we define the objective function as the difference of the two terms:(6)

To optimize this objective function, we employ a first-order incremental search [21], which builds up a set *F* starting from the empty set and at each step adding to the current *F* the edge c_*_ that maximizes:(7)

The search algorithm stops when the score for c_*_ drops below a threshold (we use 0.01 for the results shown below).

The original mRMR formulation with first-order incremental search was proved to be equivalent to max-dependency (i.e., to provide the most information about the target classification) [21]. The proof carries over to our version upon substituting our formulations of redundancy and relevance (discrete, with choices of SSE pairs providing information about models) in place of the original ones (continuous, with gene profiles representing different types of cancer or lymphoma). Essentially, it can be proved that the optimal max-dependency value is achieved when each feature variable is maximally dependent on the class of samples, while the pairwise dependency of the variables is minimized. Furthermore, this objective can be obtained by pursuing the mRMR criterion in the “first-order” incremental search (i.e., greedy) where one feature is selected at a time. Therefore we don’t need to explicitly compute the complicated multivariate joint probability, but can instead compute just the pair-wise joint probabilities. We thus have an efficient algorithm for finding an optimal set of SSE pairs to differentiate models.

#### Data interpretation

In the next section, we will describe the planning of disulfide cross-linking experiments to evaluate a given fingerprint. For now, let us assume that the form of experimental data *X* regarding a fingerprint *F* is a binary vector indicating for each edge whether or not the SSE pair was found to be in contact. Let us denote by  the set of possible binary vector values for *X*. Then the likelihood takes the joint probability over the edges, testing agreement between the observed contact state and that expected under the model:(8)

where we use the subscript to get the *i*^th^ element of the set. The naive conditional independence assumption here is reasonable, since the elements of *F_i_* (SSE contact states) depend directly on the model, and are thus conditionally independent given the model. We then select the model with the highest likelihood. (If we have informative priors, evaluating model quality, we could instead select based on posterior probabilities.)

#### Plan evaluation

In the experiment planning phase, we don’t yet have the experimental data. However, we can evaluate the potential for making a wrong decision using a given plan by computing the *Bayes error*, *∊*. If we knew which model *m* were correct and which dataset *X* we would get, we could evaluate whether or not we would make the wrong decision, choosing a wrong model *m*′ due to its having a higher likelihood for *X* than the correct model *m*. The Bayes error considers separately each case where one particular model is correct and one particular dataset results, and sums over all the possibilities. It weights each possibility by its probability—is the model likely to be correct, and if it is, are we likely to get that dataset. Thus:(9)

where Pr(*m*) is the prior probability of a model, which we currently take as uniform, but could instead be based on fold recognition scores. Here and in the following formulas we use an indicator function 1 that gives 1 if the predicate is true and 0 if it is false. So we assume each different model is correct (at its prior probability), and assess whether or not it would be beaten for each different data set (at probability conditioned on the assumed correct model). This framework thereby gives a probabilistic evaluation of how likely it is that we will make an error, in place of the usual empirical cross-validation that is performed to assess a feature subset selected for classification.

In the case of fold determination, there may not be a single best model—a number of models may in fact have the same fold, and thus be equally consistent with the experimental data. Thus in the data interpretation phase we would not want to declare a single winner, but instead would return a set of the tied-for-optimal models. In the experiment planning phase, we develop a complementary metric to the Bayes error, which we call the *expected tie ratio*, *τ*:(10)

The formula mirrors that for *∊*, but instead of counting the number of incorrect decisions, it counts the fraction of ties. Evaluating *τ* as we build up a topological fingerprint allows us to track the incremental power to differentiate folds, up to the point where we find that a set of models has the same fold and *τ* has flat-lined. The metric can readily be extended to account for sets of models whose likelihood is within some threshold of the best.

Finally, the topological fingerprint approach allows us to handle the “none-of-the-above” scenario, when we decide that no model is sufficiently good; i.e., the correct fold isn’t represented by a predicted model. While in other contexts that would be done by comparing the likelihood to some threshold (is the selected model “good enough”?), here we can actually explicitly consider the chance of not considering the correct fold. Note that since a fingerprint typically has a small number of SSE pairs, we can enumerate the space  of its possible values (indicating whether or not each SSE pair in the fingerprint is in contact). Some of those values, , correspond to models in *M*, while the rest, , are “uncovered”. We want to decide if an uncovered fold  is better than the fold *f* for the selected model. Moving from models to folds, we can evaluate Pr(*X* | *f*) by a formula like Eq. 8, simply testing whether each *X_i_* has the value specified in *f*. Then we can decide that it is “none of the above” (models) if  such that .

Moving from data interpretation to experiment planning, we can again evaluate a plan for the probability of deciding none of the above. If we think of Bayes error as the false positive rate, then we want something more like a false negative rate. We call this metric *ν*, the *expected none-of-the-above ratio*:(11)

Thus *ν* is the fraction of experimental datasets for which an uncovered fold will be better than the best covered fold. We currently do not include a prior on *X*, in order to provide a direct assessment of how many experiments could lead to a none-of-the-above decision. However, we could obtain a weighted value by estimating Pr(*X*), e.g., from the priors on the individual SSE pairs (from Eq. 1). For the same reason, we treat Pr(*f*′) as uniform over the uncovered folds *f*′, rather than evaluating it by priors on SSE pairs. Note that the formula does not include SSE pairs in (*S* × *S*) \ *F*; i.e., pairs not in the fingerprint. This is as if they contribute equally to covered and uncovered folds, and thus do not affect the outcome. In the absence of other information or assumptions about the uncovered folds, this is a reasonable (and conservative) assumption, and yields an interpretable metric.

### Cross-link selection

Once a topological fingerprint *F* has been identified, the next task is to optimize a disulfide cross-linking plan to experimentally evaluate the SSE pairs in the fingerprint. We separately plan for each SSE pair (their conditional independence was discussed in the previous section), optimizing a set of disulfide cross-link experiments (a single cross-link per experiment), such that, taken together, these cross-links will reveal whether or not the SSE pair is in contact. The overall plan is then the union of these SSE-pair plans. Thus we focus here on planning for a single SSE pair. We must account for noise and uncertainty in both the model and the actual protein, as well as for dependency among cross-links. This paper represents the first to address these issues.

Different models may place an SSE at somewhat different residues, so when planning cross-links to probe that SSE’s contacts, it is advantageous to focus on residues common to many models (and thus able to provide information about cross-linkability in those models). We define for each SSE a set of common residues that may be used in a disulfide plan. Our current implementation includes all residues that appear in at least half of the models that have that SSE. In the following, let *R* denote the common residues for a target SSE pair.

For each model *m_i_* we construct a *residue cross-link graph G_xlink_*_,_*_i_* = (*R*, *D_i_*), in which the nodes are common residues *R* and there are edges *D_i_* ⊂ *R* × *R* between possible disulfide pairs (specific to each model). We compute the *cross-linking distance* for a residue pair as the C*^β^*–C*^β^* distance, and take as edges those with distance at most 19 Å, based on an analysis of rates of disulfide formation [7,14]. Our method could be generalized to include a more detailed geometric evaluation of the likelihood of cross-linking.

#### Probabilistic model

**Figure 2:**
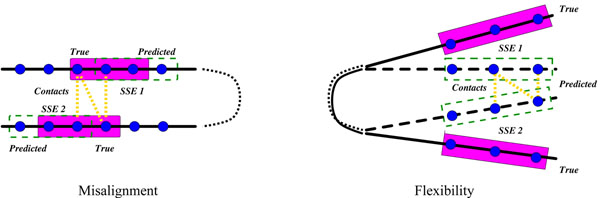
**Noise factors in cross-link planning.** Noise factors include misalignment (left) and flexibility (right). Blue dots represent residues and yellow lines their contacts. Regions in dashed lines are the modeled SSE and those in solid lines those measured by cross-linking experiments.

We must define a probabilistic model in order to evaluate the information content provided by a set of cross-links. We treat possible cross-link (pair of residues) as a binary random variable indicating whether or not there is a cross-link. We start with the model of our earlier work, in which the prior probability of a cross-link wrt a model is 0.95 for distances ≤ 9Å, 0.5 for distances between 9 and 19 Å, and 0.05 for those > 19 Å [7]. However, we also account for two important types of noise in this context: threading misalignment and structural flexibility (Fig. 2).

We place a distribution Pr(*δ*) over possible offsets by which an SSE could be misaligned in a model. That is, residue number *r* in the model is really residue *r* + *δ* in the protein, and thus a cross-link involving residue *r* + *δ* is really testing proximity to residue *r*. We use a distribution with 0.5 probability at 0 offset, decaying exponentially on both sides up to a maximum offset. Analysis of a model or the secondary structure prediction could provide a more problem-specific distribution. We currently consider each SSE separately; a future extension could model correlated misalignments resulting from threading. We sample a set of alternative backbones for a model, and place a distribution Pr(*b*) over the identities of these alternatives. While there are many ways to sample alternative structures, we currently use Elastic Normal Modes (ENMs) as implemented by *elNémo*[24], sampling along the lowest non-trivial normal mode. We set Pr(*b*) according to the amplitude of the perturbation, using a Hookean potential function derived from ENMs. Future extensions could model different aspects of flexibility, such as local unfolding events during which a cross-link may be captured.

These two factors result in dependence among possible cross-links: if an SSE is misaligned or has moved relative to the original model, all its cross-links will be affected. However, the cross-links are conditionally independent given the particular value of misalignment or backbone choice. Thus we have for any two cross-links *ℓ*, *ℓ′*:(12)

and similarly for backbone flexibility. Furthermore, misalignment and flexibility are independent.

#### Experiment planning

Our goal is to select a “good” set of residue pairs *L* ⊂ *R* × *R* to experimentally cross-link, in order to assess whether or not the SSE pair is in contact. This is another feature subset selection problem, and we again employ an mRMR-type incremental algorithm. Here a possible cross-link *ℓ*’s relevance is evaluated in terms of the information it provides about whether or not the SSE pair is in contact: *I*(*ℓ*, *c*), where *c* is the binary random variable for contact of a target SSE pair. Redundancy is again evaluated in terms of mutual information. Thus the objective is:(13)

and we incrementally select cross-links to maximize the difference in relevance regarding contact and average redundancy with already-selected cross-links.

#### Data interpretation

Once we have experimentally assessed cross-link formation for each selected residue pair, we can evaluate the probability of the SSE pair being in contact. Let *Y* be the set of cross-linking data, indicating for each residue pair in *L* whether or not a disulfide was detected. To decide whether or not c is in contact, we will compare Pr(*Y* | *c* = 1) and Pr(*Y* | *c* = 0), and take the one with higher likelihood. Intuitively, the more cross-links that are detected, the more confident we are that the SSE pair is in contact. Thus we currently employ a sigmoidal function to evaluate the likelihood:(14)

Here *k* is the number of detected cross-links in *Y*, and *k*_0_ is the minimum number of positive cross-links for us to start believing c is in contact. For example, for *c* = 1, given a default number of 10 experiments, we set *k*_0_ = 3 and the likelihoods of *c* = 1 for *k* = 0, 3, 6 are then approximately 0.05, 0.5, and 0.95, respectively. The metric could be extended to reward the broader distribution of cross-links throughout each SSE. However, in our current framework, we find that having a sufficient number of cross-links without regard to location tends to achieve that goal.

#### Plan evaluation

Finally, in order to assess an experiment plan’s robustness, we develop a Bayes error criterion to evaluate the probability of making a wrong decision regarding SSE contact:(15)

As in the previous section, we sum over the possible outcomes (here, in contact or not) and the possible experimental results (, all binary choices for cross-links in plan *L*), weighted by their probabilities, and see which yield the wrong decision. In the absence of an informative prior for c (and one that we want to use in interpreting the data), we simply use Pr(*c* = 1) = Pr(*c* = 0) = 0.5. Note that, if desired, we could use the cross-linking Bayes error as a replacement for *q* (as 1 – *∊*) in evaluating Pr(*c* = *x*). These values could be precomputed for all candidate SSE pairs, or a fingerprint could be reevaluated and perhaps modified upon evaluating its possible cross-link plan.

## Results and discussion

We demonstrate the effectiveness of our approach with a representative set of 9 different CASP targets (Tab. 1), including proteins that are all-*α*, some that all-*β*, and some that are mixed *α* and *β*. For each target, a number of high-quality models have been produced by different groups; we evaluate those of common SSE content, as described in the methods. The models vary in similarity to the crystal structure (the PDB ID indicated), which is unknown at the time of modeling and furthermore not used for experiment planning, as well as to each other (the average root mean squared deviation in atomic coordinates, RMSD, between pairs of models is indicated). Our goal is to select for each target an experiment plan to robustly determine the model(s) of the same fold as the crystal structure.

**Table 1:** Test data sets (from CASP7)

CASP ID	PDB ID	2°	AAs	Models	Av. RMSD
T0283_D1	2hh6	5*α*	97	162	17.26
T0289_D2	2gu2	5*β*	74	34	13.45
T0299_D1	2hiy	3*α*, 3*β*	91	30	15.23
T0304_D1	2h28	2*α*, 5*β*	101	26	15.76
T0306	2hd3	7*β*	95	45	14.22
T0312_D1	2h6l	2*α*, 5*β*	132	55	16.13
T0351	2hq7	5*α*	117	65	15.42
T0382_D1	2i9c	6*α*	119	196	12.79
T0383	2hnq	2*α*, 4*β*	127	59	11.61

### Topological fingerprint selection

**Figure 3:**
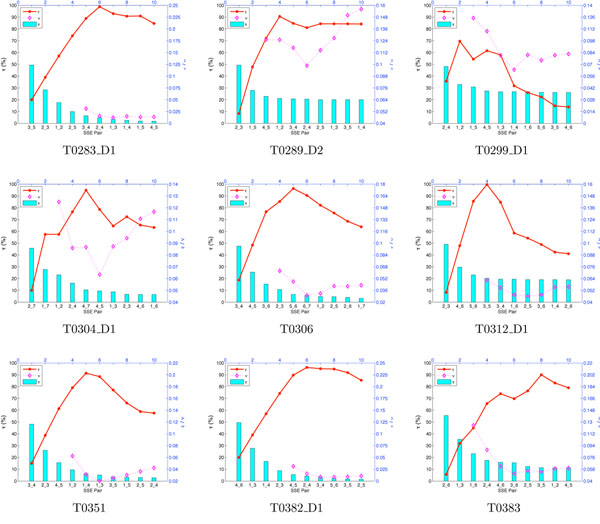
**Evaluations of fingerprints for case study targets.** The plots show Bayes error (*∊*), expected tie ratio (*τ*), and expected none-of-the-above ratio (*ν*), with addition of SSE pairs to fingerprints for targets. *x*-axis: SSE pairs. *y*-axis (left): *τ*, (%). *y*-axis (right): *∊*, *ν*.

Fig. 3 shows the trends of Bayes error (*∊*), expected tie ratio (*τ*), and expected none-of-the-above ratio (*ν*) as more SSE pairs are included in the topological fingerprint. It may seem counterintuitive that *∊* initially increases with the addition of SSE pairs. However, this is because we define the Bayes error of a tie as zero (Eq. 9), and separate out the tie ratio. With few SSE pairs in the fingerprint, *τ* is generally high—few decisions will be made, as many models look equally good, and the Bayes error is small. Then as SSE pairs are added, *τ* drops sharply—the fold is more specifically determined, decisions will be made, and the potential for error (as reflected in the Bayes error) increases. Once a sufficient number of SSE pairs has been selected, the specifically-determined fold is distinct, and the decisions are likely to be right, and *∊* will decrease. Thus it is both appropriate and helpful to consider *∊* and *τ* together, as they provide complementary information in the progress toward obtaining a unique and correct fold.

On the other hand, we observe that the *ν* value is usually 0 in the first few steps, because at that point there are not distinct folds separated, and it is easy for the SSE graphs from the predicted models to “cover” all the possible folds. *ν* becomes non-zero when there are uncovered folds. Its value first decreases because the number of covered folds and the number of uncovered folds are both increasing as more SSE pairs are included, and *ν* only gets contributions from an uncovered fold with *greater* (not equal) likelihood as the best covered fold. At some point the number of covered folds stops increasing (due to the limited set of predicted fold types), while the number of uncovered folds is still growing. Then the additional fold possibilities in the uncovered space result in a higher risk of “none-of-the-above”, and thus the *ν* value starts increasing again. This trend is particularly obvious for targets T0289_D2 and T0304_D1; in fact, we return to T0304_D1 below as a real example of “none-of-the-above”.

**Figure 4:**
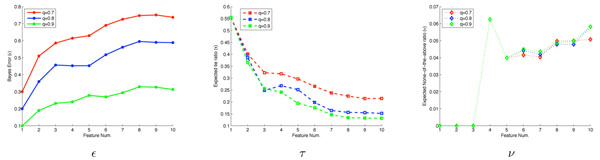
**Sensitivity analysis.** Differences in plans for target T0383, as evaluated by *∊* (left), *τ* (middle), and *ν* (right), for *q* values of 0.7 (red), 0.8 (blue), and 0.9 (green).

The fingerprint evaluation incorporates a parameter in the *q* function (Eq. 2), essentially indicating the confidence we expect to have in the experimental evaluation of an SSE pair. We performed a sensitivity analysis for three values of *q*, from 0.7 (fairly ambiguous) to 0.9 (fairly confident). Fig. 4 shows that for one target the trends are very similar for all three values; our algorithm is insensitive to the choice. Other targets display similar insensitivity (not shown).

### End-to-end simulation study

Once we have selected a topological fingerprint, we next design a disulfide cross-linking plan to determine the contact state of the selected SSE pairs. To validate the overall process (fingerprint + disulfides), we perform a simulation study. Given a selected set of residue pairs for cross-linking, we use the crystal structure (PDB entry in Tab. 1) to determine whether or not they should form disulfides (C*^β^*-C*^β^* distance < 9 Å), and treat those evaluations as the data. We also use the set of all SSE pairs to directly compare the fold of each model with that of the crystal structure, and thereby label each model as being the “correct” fold or not depending on whether or not they have the same SSE contacts for the same SSE pairs. We then evaluate whether or not the simulated data for the selected cross-linking plans result in the same conclusions as the direct comparisons of folds.

**Figure 5:**
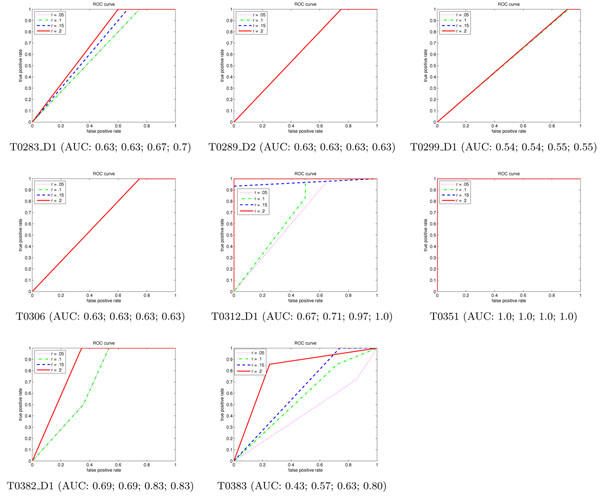
**Simulation studies.** ROC curves for eight simulation studies, at different SSE contact fraction thresholds *r*. T0304_D1 doesn’t have a predicted model that matches the crystal structure and thus is analyzed separately (see the discussion for Robustness). *x*-axis: False Positive Rate. *y*-axis: True Positive Rate. AUC: Area under the ROC curve, for *r* of 0.05, 0.1, 0.15, and 0.2 respectively.

To compare the decision based on simulated cross-linking data with that based on fold analysis, we performed a Receiver Operator Characteristic (ROC) analysis. The area under the ROC curve (AUC) measures the probability that our experiment plan will rank a randomly chosen positive instance higher than a randomly chosen negative one. The larger the AUC, the better classification power our algorithm has to detect the right fold. Fig. 5 illustrates the simulation results on eight example protein targets (ROC analysis for T0304_D1 is not applicable and we will discuss it below). ROC curves are shown for different thresholds for the percentage *r* of residues that must be in contact to declare that the SSE pair is in contact in the structure or model. A high *r* value results in very few SSE pairs deemed to be in contact (we found that to happen with *r* = 0.3), while a low one yields some fairly weak contacts. As the figure shows, a moderate *r* value of around 0.2 generally results in quite good fold determination results.

### Robustness

One of the merits of the fold determination approach is that it is robust to errors in models, and can even account for the case when none of the models is correct. The selected targets provide examples requiring such robustness; we summarize here just a couple. *Misalignment*. In Eq. 12 we account for being off by up to *δ* residues in the SSE locations. In the case of T0312_D1, there are 23 models of the correct fold, but with *δ* = 0, only 7 of them agree with the crystal structure regarding all the cross-links in the experimental plan, while with *δ* = 1 there are 14 that agree, and with *δ* = 2 there are 16. The remaining unmatched models are looser in structure, and the match is sensitive to the threshold we use to measure SSE contacts. *None-of-the-above*. For target T0304_D1, none of the models has the same SSE contact graph as the crystal structure. The GDT [6] scores of predicted models are in the low 30s, which indicates relatively poor agreement with the crystal structures. As shown in Fig. 3, the *ν* value is relatively high, indicating a potential risk of missing the right fold. Indeed once we evaluate the models under the simulated data, we find that the likelihoods are low (< 2 × 10^–3^), compared to that (≈ 0.66) of the uncovered but correct fold, which is found by enumeration.

## Conclusions

This paper presents a computational-experimental mechanism to rapidly determine the overall organization of secondary structure elements of a target protein by probing it with a planned set of disulfide cross-links. By casting the experiment planning process as two stages of feature selection—SSE pairs characterizing overall fold and residue pairs characterizing SSE pair contact states—we are able to develop efficient information-theoretic planning algorithms and rigorous Bayes error plan assessment frameworks. Focusing on fold-level analysis results in a novel approach to elucidating three-dimensional protein structure, robust to common forms of noise and uncertainty. At the same time, the approach remains experimentally viable by finding a greatly reduced set of residue pairs (tens to around a hundred, out of hundreds to thousands) that provide sufficient information to determine fold.

## Competing interests

The authors declare that they have no competing interests.

## Authors' contributions

FX, AMF, and CBK developed the approach; FX and CBK designed the algorithms, FX implemented the algorithms and collected the results; FX, AMF, and CBK analyzed the results and wrote the paper. All authors read and approved the final manuscript.
